# Doxycycline and Sitafloxacin Combination Therapy for Treating Highly Resistant *Mycoplasma genitalium*

**DOI:** 10.3201/eid2608.191806

**Published:** 2020-08

**Authors:** Duygu Durukan, Michelle Doyle, Gerald Murray, Kaveesha Bodiyabadu, Lenka Vodstrcil, Eric P.F. Chow, Jorgen S. Jensen, Christopher K. Fairley, Ivette Aguirre, Catriona S. Bradshaw

**Affiliations:** Alfred Health, Carlton, Victoria, Australia (D. Durukan, M. Doyle, L. Vodstrcil, E.P.F. Chow, C.K. Fairley, I. Aguirre, C.S. Bradshaw);; Monash University, Clayton, Victoria, Australia (D. Durukan, L. Vodstrcil, E.P.F. Chow, C.K. Fairley, C.S. Bradshaw);; Royal Children’s Hospital, Parkville, Victoria, Australia (G. Murray, K. Bodiyabadu);; The Royal Women’s Hospital, Parkville (G. Murray, K. Bodiyabadu);; Statens Serum Institut, Copenhagen, Denmark (J.S. Jensen)

**Keywords:** Mycoplasma genitalium, dual-class resistance, macrolide resistance, quinolone resistance, sexually transmitted infections, multidrug resistance, AMR, antimicrobial resistance, doxycycline, sitafloxacin

## Abstract

Antimicrobial-resistant *Mycoplasma genitalium* is becoming increasingly common and creating major treatment challenges. We present early data on combination therapy with doxycycline and sitafloxacin to treat highly resistant *M. genitalium*. We found the regimen was well tolerated and cured 11/12 infections that had failed prior regimens with moxifloxacin and pristinamycin.

*Mycoplasma genitalium* is a sexually transmitted bacterium with marked capacity for developing antimicrobial resistance (*1*). Macrolides and 4th-generation fluroquinolones, such as moxifloxacin, have been the main agents displaying efficacy against *M. genitalium*. However, macrolide resistance has increased to >50% in many nations, and quinolone resistance is increasing (*2*–*6*). In Australia, 16% of *M. genitalium* strains are reported to have dual-class resistance (*5*), and Japan reports dual-class resistance of up to 25% (*2*), resulting in infections that often cannot be cured with current recommended therapies. 

Sequential monotherapy with doxycycline followed by moxifloxacin (*7*–*9*) is currently first-line therapy for macrolide-resistant *M. genitalium* in guidelines in Australia and the United Kingdom and achieves cure in 92% of cases (95% CI 88.1%–94.6%) at our service (*7*). When the doxycycline/moxifloxacin sequential regimen fails, we use a pristinamycin-based regimen, which achieves 75% cure (95% CI 66%–82%) (*10*). Since August 2017, for patients in whom both regimens failed, we administered a combination of 100 mg doxycycline and 100 mg sitafloxacin 2 times/day for 7 days.

Access to sitafloxacin is limited in many countries, but it is available in the Asia-Pacific region. Most publications on sitafloxacin are from Japan, where its use as a monotherapy is reported to cure ≈90% of *M. genitalium* infections (*11*). However, combination therapies can optimize cure and prevent further resistance in bacteria prone to developing resistance, such as *M. genitalium*. In vitro, a combination of doxycycline and sitafloxacin (doxycycline+sitafloxacin) shows synergy for quinolone-susceptible *M. genitalium* strains but has not been evaluated for highly resistant strains (J.S. Jensen, unpub. data). We provide early data on the efficacy and tolerability of a 7-day doxycycline+sitafloxacin combination therapy for treatment-resistant *M. genitalium*. The ethics committee of Alfred Hospital (Melbourne) approved this study (approval no. 232/16).

## The Study

The study included 12 cases of macrolide-resistant *M. genitalium* detected among all patients assessed with the clinical protocol at Melbourne Sexual Health Centre (MSHC), Carlton, Victoria, Australia, for routine *M. genitalium* testing (*7*,*9*) during August 2017–April 2019 (Figure). During the study period, 96 (8%) of *M. genitalium* cases failed to respond to doxycycline/moxifloxacin; we subsequently treated 56 with pristinamycin, which failed in 15 (27%) patients. All 15 opted for combination therapy; 11 provided a test of cure, and the other 4 did not complete follow up. One additional patient also received combination therapy because her pelvic inflammatory disease (PID) did not respond to moxifloxacin. Our final analysis included 12 patients. 

**Figure:**
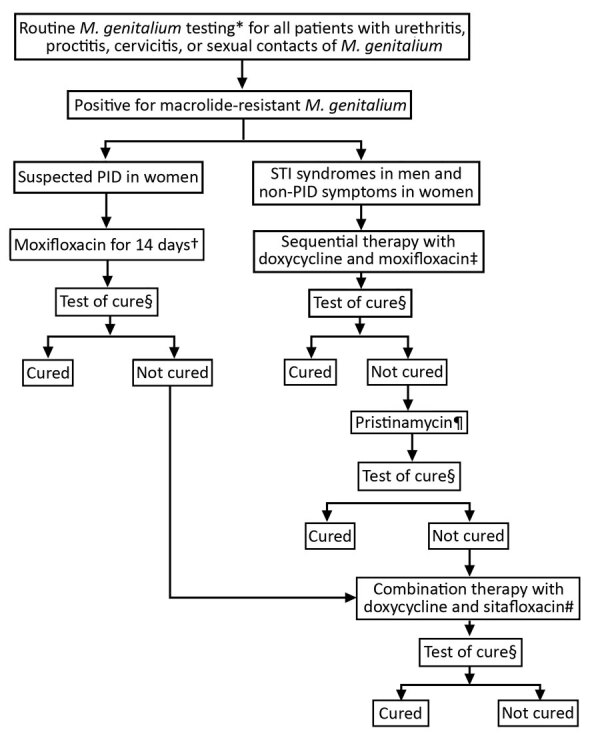
Clinical approach and treatment for patients with diagnosed macrolide-resistant *Mycoplasma genitalium* at Melbourne Sexual Health Centre, Australia. PID, pelvic inflammatory disease; STI, sexually transmitted infection. *Routine testing with the ResistancePlus MG assay (SpeeDx, https://plexpcr.com). †Moxifloxacin 400 mg/day for 14 days. ‡Doxycycline 100 mg 2 times/day for 7 days, then moxifloxacin 400 mg/d for 7 days. §Test of cure was recommended 14–28 days after completing antimicrobial treatment and all patients received a reminder. ResistancePlus MG Assay was used for all tests of cure. ¶When sequential therapy failed, patients were given a pristinamycin-based regimen for 10 days, either 1 g 4 times/day alone or 1 g 3 times/day in combination with doxycycline 100 mg 2 times/day. Doxycycline pretreatment also was given to some patients. #Doxycycline 100 mg and sitafloxacin 100 mg taken together 2 times/day for 7 days.

Among study participants, 9 sought treatment for urogenital symptoms and 1 for PID; 2 were asymptomatic contacts of persons with *M. genitalium*. Median age was 29 years (interquartile range [IQR] 27–32 years). All men (10/12) had urethral infections; the 2 women had cervicovaginal infections. 

We tested patient samples using the ResistancePlus MG Assay (SpeeDx, https://plexpcr.com). We defined treatment-resistant *M. genitalium* as microbiological failure and persistent symptoms after first-line and second-line therapies failed. We classified microbial cure as *M. genitalium* not detected and microbial failure as a positive result on test of cure 14–28 days after completing antimicrobial drug therapy. 

At MSHC, we often give doxycycline before the main regimen to reduce *M. genitalium* load and optimize cure (*12*). We term moxifloxacin-containing regimens as first-line, pristinamycin-containing regimens as second-line, and combination therapy as third-line (Table 1). All 12 patients received a moxifloxacin-based regimen; 9 had sequential doxycycline/moxifloxacin in keeping with clinical guidelines (*9*). The other 3 had moxifloxacin alone, 1 for PID, and 2 received treatments prior to coming to MSHC (1 was treated for 10 days and the other for 30 days, but we do not know the physician’s rationale for treatment duration). Moxifloxacin-based regimens failed in all patients; 11 were then treated with pristinamycin, which also failed. We administered combination therapy without preceding pristinamycin to 1 patient because of concerns regarding her PID.

**Table 1:** Antimicrobial regimens and test of cure data for patients treated for *Mycoplasma genitalium* with doxycycline and sitafloxacin combination therapy, Melbourne Sexual Health Centre, Carleton, Victoria, Australia*

Case no.	Baseline test	First-line therapy, sequential; d		TOC	Second-line therapy, sequential; d		TOC	Third-line therapy, combination; d		TOC
		Doxy†	Moxi		Doxy†	Pris		Doxy†	Combination‡	
1§	+	7	7	+	7	10	+	21	7	Cured
2	+	7	7	+	7	10	+	28	7	Cured
3	+	7	7	+	7	10	+	21	7	Cured
4§	+	None	10¶	+	7	10	+	None	7	Cured
5	+	7	7	+	7	10	+	None	7	Cured
6§	+	None	30¶	+	21	10	+	14	7	Cured
7	+	7	7	+	None	10	+	7	7	Cured
8	+	7	7	+	None	10	+	7	7	Cured
9	+	7	7	+	7	10	+	7	7	Failed
10§	+	7	7¶	+	None	10	+	3	7	Cured
11§	+	None	14#	+	None**	None	None	5	7	Cured
12	+	7	7	+	10††	10	+	3	7	Cured

*Doxy, doxycycline; Moxi, moxifloxacin; Pris, pristinamycin; TOC, test of cure; +, macrolide-resistant *M. genitalium* detected. †Doxycycline 100 mg 2 times/day commonly was given first as monotherapy in first, second, and third drug regimens; duration is specified for each case. ‡Sitafloxacin 100 mg 2 times/day and doxycycline 100 mg 2 times/day were given concurrently for 7 days.  §Patients who had prior failed antimicrobial therapy for *M. genitalium* infection before coming to Melbourne Sexual Health Centre (MSHC). Case no. 1 received 1 g azithromycin before coming to MSHC and this regimen failed. Case no. 4 received moxifloxacin 400 mg/d for 10 days before coming to MSHC and this regimen failed. Case no. 6 received 3 1-g doses of azithromycin given on separate occasions and this regimen failed, then received 2 courses of doxycycline 100 mg 2 times/day for 14 d each which also failed; then received a 30-day course of moxifloxacin 400 mg/d which also failed, before coming to MSHC. Case no. 10 received doxycycline 100 mg 2 times/day for 7 d then 1 g azithromycin which failed; then received doxycycline 100 mg 2 times/day for 7 d which failed, after which the patient received moxifloxacin 400 mg/d for 7 d, which also failed before coming to MSHC. Case no. 11 received doxycycline and azithromycin at unspecified doses or duration before coming to MSHC. ¶Moxifloxacin-containing regimen given to a patient in the community before they came to MSHC. This regimen often varied from firstline therapy given at MSHC.  #Moxifloxacin 400 mg/d × 14 d was given as a first regimen to this patient because of diagnosed pelvic inflammatory disease.  **Patient was diagnosed with possible *M. genitalium–*related pelvic inflammatory disease and did not receive a pristinamycin–containing regimen (*14*).  ††Patient was given 1 g pristinamycin 3 times/day in combination with doxycycline 100 mg 2 times/day rather than 1 g 4 times/day because both regimens have shown equivalent efficacy at our service (*10*).

All patients received doxycycline+sitafloxacin combination therapy for 7 days; 9 had preceding doxycycline for varying durations (Table 1). Among 12 patients, 11 (91.7%; 95% CI 64.9%–98.5%) were cured and achieved complete symptom resolution after combination therapy. Combination therapy failed in 1 patient who experienced persistent dysuria. Median time to test of cure after combination therapy was 20 (IQR 14–24) days. Median duration from first *M. genitalium* diagnosis to cure was 125 (IQR 106–144) days. Before test of cure, all patients were classified as no- or low-risk for reinfection by the treating clinician on the basis of no sex or 100% condom use with any partner or sex with a fully treated partner in the interval between treatment and test of cure (*7*,*12*).

All patients whose first-line and second-line therapies failed were symptomatic, including the 2 who initially were asymptomatic contacts. All 10 men reported persistent fluctuating dysuria, 6 reported urethral discharge, 2 urethral irritation or itching, and 1 meatal inflammation. Both women reported fluctuating abnormal vaginal discharge, 1 reported intermittent dysuria, and the patient with PID reported persistent dyspareunia. Men typically experienced a stepwise reduction in urethral symptoms after commencing antimicrobial drugs, but dysuria re-emerged during follow-up.

Among 10 patients for whom adherence and adverse effects are available, 9 (90.0%; 95% CI 60.0%–99.5%) reported taking all doses of both drugs, including the patient whose treatment failed; 1 reported missing 1 tablet of sitafloxacin. Six (60.0%; 95% CI 31.3%–83.2%) patients reported no adverse effects. Among the other 4, adverse effects were mild and resolved spontaneously (1 each of diarrhea, arthralgia, tendon pain, and possible blurred vision).

Sanger sequencing of the quinolone resistance–determining regions of the *parC* and *gyrA* genes revealed single nucleotide polymorphisms for *parC* in all cases and for *gyrA* in 5/12 cases before combination therapy (Table 2). The *parC* mutations corresponded to amino acid changes S83I (G248T; n = 11) and D87N (G259A; n = 1). The *gyrA* mutations corresponded to amino acid changes M95I (G285A; n = 3), A79S (G235T; n = 1), and D99N (G295A; n = 1). In 1 case, a *gyrA* mutation appeared to develop after moxifloxacin failure (case 9; Table 2). The patient in whom combination therapy failed had a single *parC* S83I change detected (Table 2).

**Table 2:** Amino acid changes in the quinolone resistance–determining regions of *parC* and *gyrA* genes of macrolide-resistant *Mycoplasma genitalium* in patients treated with combination therapy, Melbourne Sexual Health Centre, Carlton, Victoria, Australia*

Case no.	Sexual orientation	Baseline test			TOC after first-line therapy			TOC after second-line therapy			TOC after third-line therapy	
		*parC*	*gyrA*		*parC*	*gyrA*		*parC*	*gyrA*		*parC*	*gyrA*
1	MSM	NA	NA		S83I	n/A		S83I	D99N		Cured	
2	MSM	S83I	M95I		S83I	M95I		S83I	M95I		Cured	
3	MSM	S83I	WT		S83I	WT		S83I	WT		Cured	
4	MSW	NA	NA		NA	NA		S83I	A79S		Cured	
5	MSW	D87N	WT		D87N	WT		D87N	WT		Cured	
6	MSW	NA	NA		S83I	WT		S83I	WT		Cured	
7	MSW	S83I	WT		S83I	WT		S83I	WT		Cured	
8	MSM	S83I	WT		S83I	WT		S83I	WT		Cured	
9	MSW	S83I	WT		S83I	WT		S83I	WT		S83I	WT
10	MSM	NA	NA		S83I	WT		S83I	WT		Cured	
11	W	S83I	WT		S83I	M95I		ND	ND		Cured	
12	W	S83I	M95I		S83I	M95I		S83I	M95I		Cured	

*MSM, men who have sex with men; MSW, men who have sex with women only; NA, sample was not available for sequencing; ND, not done; W, heterosexual woman; WT, wild type.

## Conclusions

Combination therapy with doxycycline+sitafloxacin was well tolerated and effective against treatment-resistant *M. genitalium*. The regimen was acceptable to clinicians and is now used as our third-line regimen.

 Moxifloxacin failure has been associated with specific polymorphisms in the quinolone resistance–determining regions of *parC* (*2*,*5*). The *parC* G248T mutation, which causes amino acid change S83I, is the most common mutation associated with moxifloxacin failure (*5*). The less common G259A(D87N) mutation has been associated with higher moxifloxacin MICs in 3 *M. genitalium* strains (J.S. Jensen, unpub. data). S83I contributes to both sitafloxacin and moxifloxacin failure (*5*). Although sitafloxacin is more likely than moxifloxacin to cure an infection carrying an S83I mutation, we previously found concurrent *gyrA* mutations, particularly M95I, increased the risk for sitafloxacin failure (*5*). In this study, moxifloxacin failed in all 12 cases with a *parC* mutation and 5 had a concurrent *gyrA* mutation. However, 11/12 cases were cured with the doxycycline+sitafloxacin combination. Of note, the 1 treatment failure occurred in a case with only the *parC* G248T/S83I mutation, no concurrent *gyrA* mutation, and no more detectable resistance than cured cases.

Of note, sitafloxacin alone might have cured some or all infections. Further studies comparing sequential and combination therapy with doxycyline+sitafloxacin in highly resistant *M. genitalium* are needed. However, treatment failures, particularly in cases with concurrent *parC* and *gyrA* mutations, would be expected. Moreover, the variable duration of preceding doxycycline may have improved cure. 

In conclusion, our results provide important early data on the efficacy and tolerability of doxycycline+sitafloxacin combination therapy to cure highly resistant *M. genitalium* infections. This approach could become part of a broader stewardship strategy to evaluate combination therapy, which might be needed to further prevent development of antimicrobial-resistant *M. genitalium*.
